# Supplementary material for: The impact of pelvic lateral rotation on hindlimb kinematics and stride length in the red-legged running frog, Kassina maculata
Source: R Soc Open Sci. 2019 May 8;6(5):190060. doi: 10.1098/rsos.190060 (PMC6549954; doi:10.1098/rsos.190060)
Supplement: Supplementary Material [file rsos190060supp1.docx]

Supplementary Material:

**Table S1: Linear Mixed Model Statistical Analyses from R Code Output.**

Values presented to 3 decimal places. There were 23 observations for 8 groups for all analyses. Individual frog set as random effect for all cases. Degrees of freedom = 14 in all cases.

SD, standard deviation; S.E, standard error.

| **Fixed Effects** | **Statistical Analyses** | | | | | | | | | | |
| --- | --- | --- | --- | --- | --- | --- | --- | --- | --- | --- | --- |
|  | *Intercept SD* | *Residual SD* | *Intercept* | | | | *Gradient* | | | | *Ω^2^* |
|  |  |  | *Value* | *S.E.* | *t-value* | *P-value* | *Value* | *S.E.* | *t-value* | *P-Value* |  |
| Speed vs. Pelvic Angle | 2.166 | 2.711 | 19.391 | 2.242 | 8.650 | <0.001 | -48.507 | 15.30 | -3.170 | 0.007 | 0.706 |
| Pelvic Angle  vs. TMT Gain | 0.884 | 0.813 | -0.181 | 0.850 | -0.213 | 0.834 | 0.309 | 0.059 | 5.247 | <0.001 | 0.843 |
| Pelvic Angle vs. Ankle Gain | 0.811 | 0.676 | 0.799 | 0.734 | 1.088 | 0.295 | 0.150 | 0.050 | 2.985 | 0.010 | 0.774 |
| Pelvic Angle vs. Knee Gain | 0.763 | 0.588 | 0.272 | 0.658 | 0.413 | 0.686 | 0.217 | 0.045 | 4.870 | <0.001 | 0.861 |
| Speed vs. Stride Length | 0.004 | 0.005 | 0.071 | 0.005 | 15.281 | <0.001 | 0.029 | 0.032 | 0.904 | 0.381 | 0.408 |
| Speed vs. Stride Frequency | 0.094 | 0.141 | 0.146 | 0.109 | 1.336 | 0.203 | 12.287 | 0.748 | 16.421 | <0.001 | 0.966 |

**
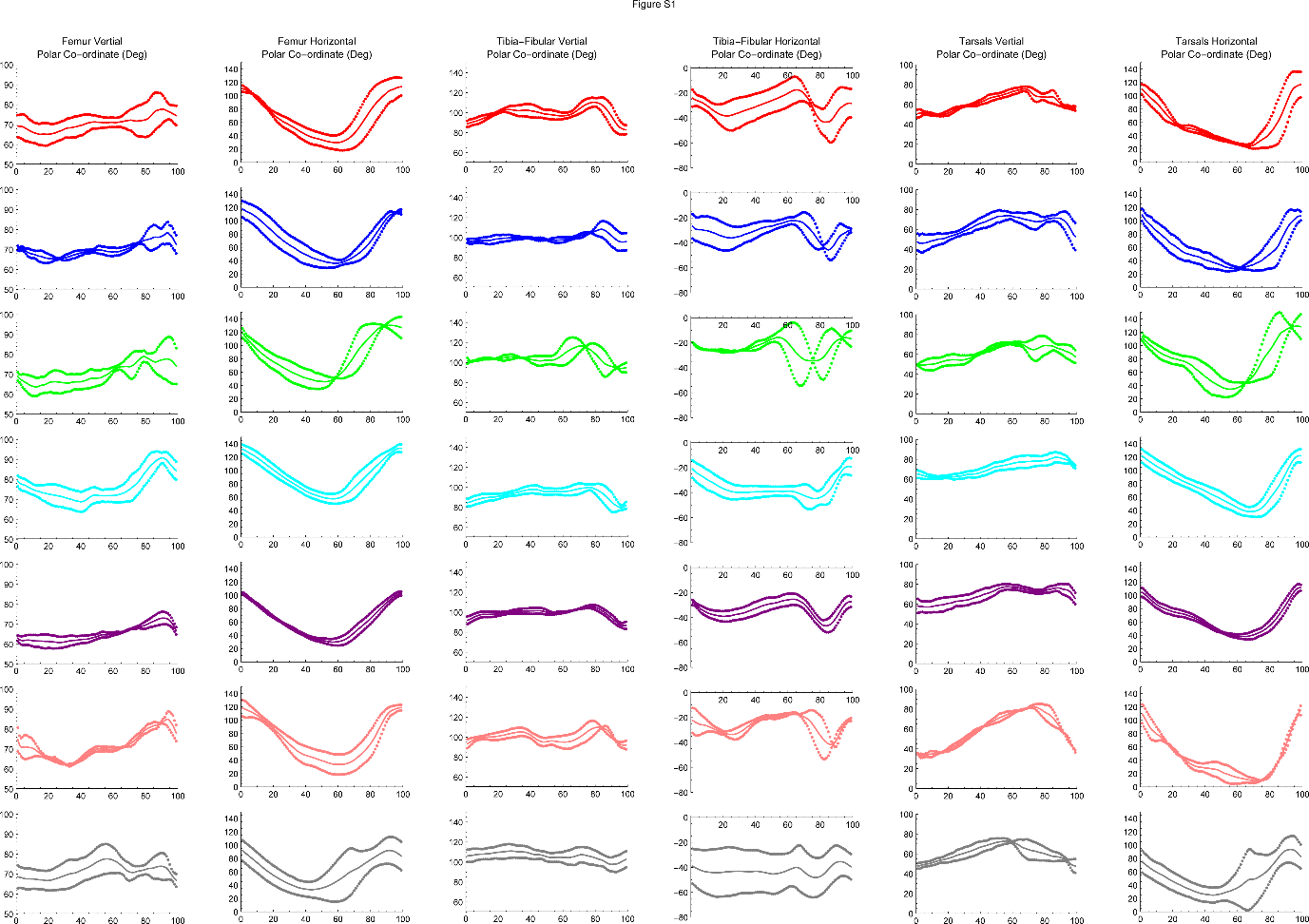
**

**Figure S1: Polar angle co-ordinate data grouped by individual frog**. Each row represents mean ±SD for 2-5 trials averaged for a given individual (data from Animal 0 (yellow) are not included here as only a single trial was collected). Individual frog colours match the colour scheme used in Figures 5 & 6 in the main document. Columns represent the horizontal and vertical components of motion for the femur, tibia-fibula and tarsals. Note that the axes have been scaled across columns to make the patterns clear.
